# Not Only Toxic but Repellent: What Can Organisms’ Responses Tell Us about Contamination and What Are the Ecological Consequences When They Flee from an Environment?

**DOI:** 10.3390/toxics8040118

**Published:** 2020-12-12

**Authors:** Cristiano V. M. Araújo, Abdelmourhit Laissaoui, Daniel C. V. R. Silva, Eloisa Ramos-Rodríguez, Enrique González-Ortegón, Evaldo L. G. Espíndola, Francisco Baldó, Freylan Mena, Gema Parra, Julián Blasco, Julio López-Doval, Marta Sendra, Mohamed Banni, Mohammed Ariful Islam, Ignacio Moreno-Garrido

**Affiliations:** 1 Department of Ecology and Coastal Management, Institute of Marine Sciences of Andalusia (CSIC), Puerto Real, 11519 Cadiz, Spain; quique.gonzalez@icman.csic.es (E.G.-O.); julian.blasco@csic.es (J.B.); ignacio.moreno@icman.csic.es (I.M.-G.); 2 National Centre for Nuclear Energy, Science and Technology, BP 1381, Rabat RP 10001, Morocco; laissaoui@cnesten.org.ma; 3 Institute of Xingu Studies, Federal University of Southern and Southeastern Pará, São Félix do Xingu, PA 68507-590, Brazil; daniel.clemente@unifesspa.edu.br; 4 Department of Ecology and Institute of Water Research, University of Granada, 18010 Granada, Spain; eloisa@ugr.es; 5 NEEA/CRHEA/SHS, São Carlos Engineering School, University of São Paulo, Av. Trabalhador São Carlense, 400, São Carlos, SP 13.560-970, Brazil; elgaeta@sc.usp.br; 6 Instituto Español de Oceanografía (IEO), Centro Oceanográfico de Cádiz, 11006 Cádiz, Spain; francisco.baldo@ieo.es; 7 Regional Institute for Studies on Toxic Substances (IRET), Universidad Nacional, 86-3000 Heredia, Costa Rica; freylan.mena.torres@una.cr; 8 Animal Biology, Plant Biology and Ecology Department, University of Jaén, 23071 Jaén, Spain; gparra@ujaen.es; 9 Catalan Institute for Water Research (ICRA), Scientific and Technological Park of the University of Girona, H2O Building, C/Emili Grahit, 101, 17003 Girona, Spain; jclopez@icra.cat; 10 Faculty of Sciences, University of Girona, Campus de Montilivi, 17003 Girona, Spain; 11 Institute of Marine Research (IIM), National Research Council (CSIC), Eduardo Cabello 6, 36208 Vigo, Spain; msendra@iim.csic.es; 12 Laboratory of Biochemistry and Environmental Toxicology, Higher Institute of Agronomy, 1349-017 Chott-Mariem, Tunisia; m_banni@yahoo.fr; 13 Department of Aquatic Resource Management, Faculty of Fisheries, Sylhet Agricultural University, Sylhet 3100, Bangladesh; arif19df1.sau@gmail.com

**Keywords:** avoidance, behavior, habitat selection, multi-compartmented systems, non-forced exposure, repellency

## Abstract

The ability of aquatic organisms to sense the surrounding environment chemically and interpret such signals correctly is crucial for their ecological niche and survival. Although it is an oversimplification of the ecological interactions, we could consider that a significant part of the decisions taken by organisms are, to some extent, chemically driven. Accordingly, chemical contamination might interfere in the way organisms behave and interact with the environment. Just as any environmental factor, contamination can make a habitat less attractive or even unsuitable to accommodate life, conditioning to some degree the decision of organisms to stay in, or move from, an ecosystem. If we consider that contamination is not always spatially homogeneous and that many organisms can avoid it, the ability of contaminants to repel organisms should also be of concern. Thus, in this critical review, we have discussed the dual role of contamination: toxicity (disruption of the physiological and behavioral homeostasis) vs. repellency (contamination-driven changes in spatial distribution/habitat selection). The discussion is centered on methodologies (forced exposure against non-forced multi-compartmented exposure systems) and conceptual improvements (individual stress due to the toxic effects caused by a continuous exposure against contamination-driven spatial distribution). Finally, we propose an approach in which Stress and Landscape Ecology could be integrated with each other to improve our understanding of the threat contaminants represent to aquatic ecosystems.

## 1. Introduction

The concept of the risk linked to contaminants in ecotoxicology is strongly associated with the toxic effects they might produce. Therefore, the more toxic a contaminant is, the more dangerous it is [1]. Initially, this specific focus on toxicity, at the expense of a more ecological approach, was not a problem since toxicity was the driving force that drove the emergence of ecotoxicology [2]. However, it should not be the unique focus. The inclusion of more ecological approaches, beyond just toxicity, has long been called for [1,3,4,5,6,7,8]. This need to integrate ecological concepts into ecotoxicology has, in fact, led to newer approaches such as Stress Ecology (and its subdomain Chemical Stress Ecology): the study of contamination-driven alterations to biological systems [9,10]. According to these authors, this approach should cover not only the effects at the individual level, considering the entire life cycle, but also the intra- and interspecies interactions as well as their relationship with the environment [6]. Possibly, this historic trend of ecotoxicologists to apply a more toxicological approach instead of moving towards ecology comes from the origin of this science, initially defined as a branch of toxicology, due to the relatively few ecologists working in this area [2]. However, other approaches are emerging in ecology and include different stressors to study their effects when acting simultaneously on biota. Undoubtedly, information about the toxicity of chemicals for as many species as possible is crucial for environmental risk assessments (ERAs), but a more ecological view that would broaden the perspective of contaminant-driven environmental damage is urgently required [1,11,12].

The ecotoxicological approaches with the most ecological implications are mainly based on indirect effects (reaching the higher levels of biological organization) and seek to cover broader spatial scales, for instance: the structure and functioning of ecosystems (including the concepts of functional redundancy, resistance and resilience), metapopulation and community ecology, landscapes in spatially connected and heterogeneous (patchy) environments, (re)colonization, ecosystem functions and services, etc. [1,5,13,14,15,16,17,18]. Although it is widely known that organisms select their place to live according to their limits of tolerance, food availability, mating success, protection from predators and etc., under this ecological umbrella, the capacity of contaminants to repel organisms and modify their behavioral fitness and spatial distribution is a subject that should be taken into account (see the reviews by De Lange et al. [7]; Araújo et al. [19], Araújo and Blasco [20] and Moreira-Santos et al. [11]), mainly as an early warning signal [21]. The concept of repellency in ecotoxicology is linked to the avoidance behavior triggered by chemicals under conditions in which organisms are given multi-choice experiments, containing at least two chemically different environments [22,23,24,25,26]. The possibility of simulating scenarios in which organisms can move freely among chemically different environments allows us to assess any differences in the level of repellency of the contaminants and understand how this repellency drives the spatial distribution of organisms [27,28]. This approach changes the focus of the effects of the contaminants from toxicity to concepts related to dispersion, migration, and habitat selection processes [18,29]. Although no effect is expected to occur on individuals (avoiders might only be in contact with the contaminant for a very short time), the migration of part of or even the entire population could be considered just as disastrous as the death of the organisms at the local scale [18,21,30]. Even a partial disappearance of populations might cause a reduction in biodiversity, affect the ecosystem’s structure and functionality as well as its resilience, and the capacity to withstand other stressors (e.g., environmental changes and other anthropogenic impacts) [6,18,31]. Therefore, the environmental disturbance caused by contamination should also include the way in which chemicals repel organisms, changing their habitat selection processes and then their spatial distribution patterns. Another important mechanism used by many planktonic invertebrates (e.g., cladocerans, copepods, ostracods, and rotifers) to escape stressful conditions is temporal avoidance by entering dormant stages [32,33]. This adaptation allows species: to remain in highly unpredictable and variable environments, favors the dispersion to, and colonization of, new habitats and provides higher resilience to the ecosystem [32,33,34,35]. In spite of the importance of this adaptive mechanism and the little knowledge of its role in contaminated environments [36], the current review is exclusively focused on spatial avoidance (repellency).

**Figure 1:**
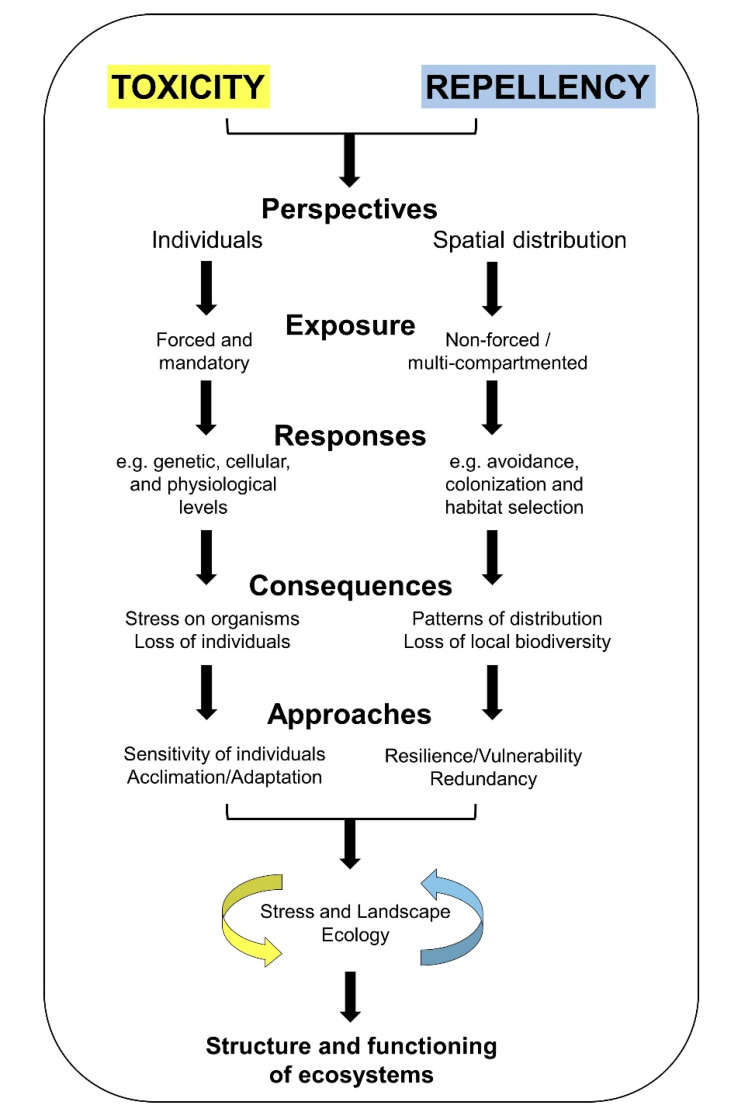
A schematic representation of some concepts linked to toxicity (defined according to the traditional forced exposure approach) and repellency (defined according to the non-forced multi-compartmented exposure approach) that could be integrated to understand the effects of contamination on the structure and functioning of ecosystems better. Regarding the toxicity approach, the scheme shows that the main perspective of toxicity is focused on individuals, in which the forced exposure is the more traditional exposure method. From this perspective, some classes of responses at different biological levels, the effects expected (from stress to loss of species) and the concepts that the studies focus on (sensitivity of species as well as possible mechanisms of acclimation and adaptation to face contamination) are represented. Regarding the repellency, the perspective is focused on the spatial distribution of organisms based on a non-forced exposure (as individuals are not mandatorily exposed), considering the responses related to the dispersion of species, whose effects might only be perceived due to changes in the spatial distribution of the species and possible loss of local biodiversity. The main approaches to be dealt with in the repellency-based approach include the ecosystem’s capacity to resist or become more vulnerable to the changes depending on the redundancy of species (avoiders will be replaced by non-avoiders with similar or different functions). Finally, the integration of both approaches makes it possible to apply a broader approach that includes Stress and Landscape Ecology.

The repellent character of a substance is probably not necessarily directly related to its toxicity, and so a highly repellent contaminant could have a low toxicity. In fact, in some cases a biphasic response (initial attraction at low concentrations and avoidance at higher concentrations), described as behavioral hormesis, has been observed [37,38]. The aim of the current critical review is to present a discussion on the avoidance response of organisms to escape from continuous exposure and the ecological consequences of this response compared to the traditional approach based on the toxic effects of the contaminants. The discussion focuses on the dichotomy between toxicity and repellency (Figure 1), considering their major differences, both methodological (forced exposure against non-forced multi-compartmented exposure systems) and conceptual (individual stress due to the toxic effects caused by a continuous exposure against contamination-driven spatial distribution). Regarding the exposure approach to assess repellency, we have exclusively focused on non-forced multi-compartmented exposure systems because they are a more complex method capable of simulating environmental heterogeneity, either as gradients or patches of contamination [22,30,39]. Secondly, a brief comparison between the repellency and toxicity of some chemicals is provided. Finally, we discuss the ecological implications of avoidance in multi-compartmented systems and the conceptual improvements that this approach might provide to ERAs in the light of spatial displacement (extinction at the local level, re-colonization of environments, chemical fragmentation of habitats and habitat connectivity, metapopulation, metacommunity, and meta-ecosystem). A summarized schematic representation of the concepts discussed in the current review, as well as the advantages of integrating toxicity and repellency in the environmental risk studies is shown in Figure 1. Briefly, *Toxicity* refers to the stress directly affecting the individuals with the consequent loss of (from genetic to behavioral) homeostasis due to their sensitivity or by provoking acclimation or adaptation. On the other hand, *Repellency* is here considered an indirect effect, due to the absence of damage (at any level) on individuals, as the exposure is not continuous and the response is based on the capacity of organisms to perceive contamination and avoid it: the displacement towards another area indicates the potential aversive nature of the contaminated habitat, but not a toxic effect on individuals. In this case, the loss of biodiversity at the local scale might produce problems within the ecosystems related to vulnerability and functional redundancy. The methodological differences in relation to exposure systems (forced and mandatory exposure against non-forced and multi-compartmented exposure) determine the conceptual differences between focusing on toxicity or repellency. Both approaches applied concomitantly might contribute to the integration of Stress Ecology with Landscape Ecology.

## 2. Toxicity: The Traditional Ecotoxicological Response

The main role attributed to ecotoxicology since it was launched as a science has been to provide evidence concerning the potential toxic effects of chemicals on organisms [2,40,41]. To employ a vision beyond traditional toxicology (effects of contaminants on a particular species with the aim of protecting humans), ecotoxicology has attempted to focus on effects at different levels of biological organization, from sub-organisms to community (sometimes making inferences about an ecosystem’s structure and functioning) [1,5,18]. Thus, ecotoxicology is a tool to complement the information of ERA studies, previously based on chemical and ecological data. Due to this role in ERAs, ecotoxicology has begun to develop a very important legal role, which has required the standardization and regulation of procedures. Therefore, although an environmentally more realistic laboratory-scale was always desired, ecotoxicological assays moved towards prioritizing other features rather than the ecological relevance of the experiments, for instance: easy development, practicability, cost-effectiveness, and replicability [42,43]. In this sense, ecotoxicological assays progressed towards a standard method that consists in exposing organisms to different concentrations of a chemical (or environmental samples such as water and sediment) and, after a previously established exposure period, some responses/endpoints are measured and compared with a control (unexposed) population [10,44]. Throughout the exposure period, the organisms are mandatorily in continuous contact with the contaminant, allowing a direct concentration-response relationship to be established. Therefore, regardless of the level of observation, whether at the sub-individual level or higher, this type of exposure (forced and mandatory exposure) means the effects are specifically linked to toxicity.

Although the forced exposure is a standard approach used in almost all ecotoxicological studies, the endpoints employed to measure the potential toxic effect of a chemical have been described from different biological organization levels and perspectives: biochemical, cellular, molecular, physiological (e.g., growth, feeding), histopathological, and behavioral effects [1,45,46]. Whether at a low or high biological level, the toxicity comes from a cascade of events that begin with the absorption and/or adsorption of the contaminants and the consequent impairments/disruptions they may produce. This approach has helped to detect the contaminants with a very high risk to the environment due to their toxicity and to identify highly susceptible species within the various ecosystems studied. This information has been useful for environmental conservation, not only for scientists, but also for regulatory enforcement. However, when organisms are confronted with contaminants, it should be considered that three different reactions can occur: conformity, regulation, or avoidance [47]. The use of a forced exposure approach includes the conformity and the ability to regulate the contaminants, but it does not comprise the possibility of escaping. A forced exposure environment assumes that environments are chemically homogeneous and that there is no option to avoid exposure. This assumption has recently changed with the development of non-forced multi-compartmented exposure systems [30]. An avoidance behavior is no longer assessed exclusively based on changes in the swimming patterns, but rather on dispersion within a chemically heterogeneous environment. Therefore, answers to questions like “what if aquatic animals move away from contaminated habitats before suffering adverse physiological effects?” [11] seem to be easier to provide now.

## 3. Avoidance: A Repellency-Driven Behavioral Response

**Figure 2:**
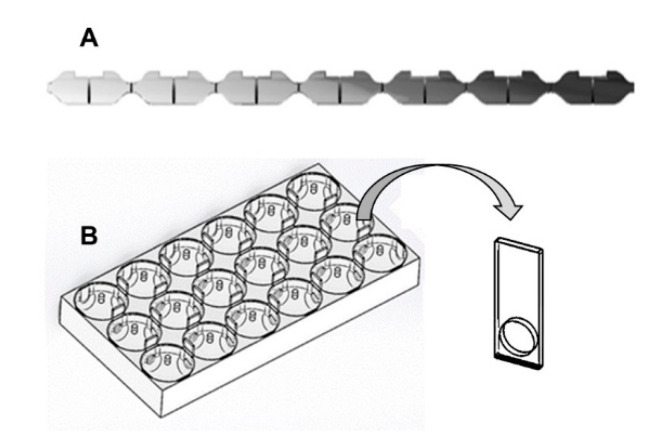
Schematic representation of the two most widely used non-forced multi-compartmented exposure systems in avoidance experiments: (**A**): linear system representing a contamination gradient indicated by the scale of grey and (**B**): HeMHAS (Heterogeneous Multi-Habitat Assays System) with the gate used to open or close the connections between compartments in all directions.

Traditionally, avoidance has been linked to behavioral changes, such as overexcitement or lethargy, that could indicate a response to flee or not from contaminants [47,48,49,50]. Since this assumption is based on a forced exposure approach, it does not allow us to know whether organisms could discriminate among different concentrations in a smoothly heterogeneous scenario, rather than only in an abruptly modified chemically heterogeneous environment. The selection of the studies under discussion in this section was based on whether they were performed in multi-compartmented exposure systems (see examples of the most used systems in Figure 2). Although many different non-forced systems can provide a contamination gradient for organisms [22], multi-compartmentalization allows the magnitude of the avoidance response to be related to all the concentrations (or water and sediment samples from different origins) used to make up the gradient. Then, a typical concentration-response can be obtained. This also favors the comparison on how sensitive avoidance is in relation to the data with other endpoints when comparing LC*x* or EC*x* (lethal or effective concentration to *x*% of the population) values with AC*x* values (concentration eliciting an avoidance of *x*% of the population).

The development of non-forced systems has provided the possibility of confronting organisms with different scenarios to identify the more attractive or repellent zones. A pioneer flow-through multi-compartmented system, in which a smooth linear gradient (1D) of contamination can be simulated, was developed by Lopes et al. [30]. This system was later simplified by Rosa et al. [51], who turned it into a static multi-compartmented system. In recent years, a more complex system (HeMHAS—Heterogeneous Multi-Habitat Assay System) has been proposed by Araújo et al. [52]. Both systems have been used in studies with different organisms and chemicals. Although bi-compartmented exposure systems (two choice options) are also widely used to assess repellency (see review by Jutfelt et al. [22]), we briefly present data from multi-compartmented exposure systems in this section due to their ecological relevance and environmental complexity in terms of the concepts discussed here. Detailed information can be obtained in reviews by Araújo et al. [19], Araújo and Blasco [20], and Moreira-Santos et al. [11]. All comparisons with other endpoints should be made with caution, since avoidance is usually measured after a very short exposure time (between 3 and 12 h, depending on the exposure system and the maintenance of the contamination gradient).

The first evidence of avoidance in a multi-compartmented system was described for the cladoceran *Daphnia longispina* [30]. These authors observed that among the different lineages tested, the sensitivity and early reactiveness of the organisms to avoid copper was directly related to the lethal sensitivity of the lineages. Other invertebrates such as the cladoceran *D. magna* (exposed to pulp mill effluents [53]; atrazine [51]; and salinity as stress factor [54]), the freshwater copepod *Boeckella occidentalis intermedia* (crude oil as the contaminant [55]), the ostracod *Heterocypris incongruens* (salinity as the stress factor [54]), the gastropod *Peringia ulvae* (sediment spiked with cadmium [56]), the freshwater shrimp *Atyaephyra desmarestii* (exposure to copper [39,57,58,59]), the marine shrimp *Litopenaeus vannamei* (exposed to copper [60,61]), and the saltmarsh shrimp *Palaemon varians* (exposed to musks and sunscreens [25,62]) have been tested for avoidance. In general, the avoidance response reported in those studies was more sensitive than the lethal and some sub-lethal endpoints described by other authors (see references cited above). However, the avoidance and mortality of the copepod *B. occidentalis intermedia* was similarly sensitive [55] and the 21-day reproduction test with *D. magna* exposed to atrazine proved to be more sensitive than avoidance [51].

Regarding vertebrates, avoidance studies in multi-compartmented systems have mainly focused on amphibians and fish. Tadpoles of the amphibian *Lithobates catesbeianus* have proved to be able to avoid different chemicals such as copper [63], the fungicide pyrimethanil [64], the pesticide abamectin [65], the 2,4-dichlorophenoxyacetic acid herbicide [66], the herbicide diuron [67], and solution containing mining tailings [68]. Avoidance by tadpoles of *Leptodactylus latrans* and *Pelophylax perezi* of contamination by copper and pyrimethanil has been also described [63,64]. In almost all these studies, avoidance was shown to be a highly sensitive response when compared with lethal or even sub-lethal (e.g., development, weight, and swimming behavior) responses (see previous citations). On the other hand, in some studies avoidance was not the most sensitive response when compared with, for instance, the responses of the: feeding rate, growth rate (SVL) and weight gain rate of tadpoles of *Xenopus laevis* exposed to gold nanorods [69] and the speed and distance responses after 16 days of exposure to mining tailings [68].

The avoidance response using the multi-compartmented approach has been mainly used for two fish species: zebrafish (*Danio rerio*) and guppy (*Poecilia reticulata*). The first avoidance study in a multi-compartmented scenario with fish was performed with zebrafish that were exposed to gradients of copper and effluent from acidic mine drainage [70]. In that study, the authors attested that avoidance is a quick response, so that the avoidance observed after 12 h exposure did not vary from exposure periods of up to 96 h. This was possible mainly because the system maintained the contamination gradient for a long time. Later, in a study also performed with zebrafish exposed to the fungicide pyrimethanil, it was shown that the exposure period to measure avoidance could be as short as 4 h [71]. This is of great importance if static systems (without peristaltic pumps) are used, as it is difficult to maintain the gradient for a long time when the fish are swimming continuously. Avoidance studies with fish have also been performed with different contaminants such as: tuna fish processing plant effluent [72], triclosan [73], atrazine [74], river samples [75,76], bisphenol [77], copper [78,79], fipronil and 2,4-D [80], dairy wastewater [76], among others. In the study by Araújo et al. [71], the avoidance response was assessed during very short exposure periods, sometimes not exceeding 4 h. In almost all cases, the avoidance initially observed (e.g., after 30 min) was similar to the avoidance at different periods during the remaining hours of the experiment. Furthermore, in some of those studies, avoidance occurred at sub-lethal concentrations and even at environmentally relevant concentrations [66,73,74,77].

The use of the multi-compartmented exposure approach to assess the ability of the organism to escape from contamination seems to be a suitable alternative to understand the environmental risks caused by the repellent characteristics of the contaminants. In addition, the avoidance response has been detected after a very short exposure time, generally not superior to 12 h [11,19,70]. However, the use of avoidance in multi-compartmented systems has some limitations, since its ecological relevance is conditioned to heterogeneous environments and the motility of the species (e.g., i. only organisms with active motility and displacement ability can be used; ii. the environmental relevance of the scenario simulated depends on the chemical heterogeneity occurring in the environment; iii. the spatial scale of the scenario simulated in the laboratory is much lower than the real spatial scale; iv. the time of experimentation is determined by how long the differences among the concentrations is maintained inside the system; v. the use of bigger species requires much bigger exposure systems and a greater quantity of chemicals). The current approach does not replace the traditional forced exposure but provides a complementary tool that could be applied to better understand the potential damages that chemicals can cause, by not only focusing on toxicity, but also on repellency. In this sense, it is important to point out that repellency can be as variable as the different chemical structure of the contaminants. In fact, experimental evidence has shown that even potentially toxic chemicals can present a certain level of attractiveness to organisms rather than repellency [25,26,37,81,82].

## 4. The Higher the Toxicity, the Higher the Repellency?

Although there could be a tendency to assume that the repellency of a contaminant is related to its toxicity, this relation seems not to be linearly direct, especially for chemicals with a neurotoxic action [65,67,82]. It has been shown that potentially toxic chemicals can exert some attraction to organisms, a similar phenomenon to the classical hormesis effect that might be limited by increasing concentrations [37,38]. For instance, attraction to contamination was observed: in the mud snail *Illyanassa obsolete* and the amphipod *Corophium volutator* exposed to chlorothalonil [83] and in the crayfish *Orconectes virilis* exposed to the antidepressant sertraline [82]. In a study with essential oil extracted from the fruits of *Evodia lenticellata*, monoterpenes were shown to be the most toxic group of chemicals, but not the most repellent for the insects *Tribolium castaneum*, *Lasioderma serricorne* and *Liposcelis bostrychophila* [23]; on the other hand, caryophyllene oxide and β-caryophyllene were only moderately toxic, but strongly repellent.

For aquatic animals, such as fish and crustaceans, their interaction with the environment and their behavioral response to chemical signals are significantly mediated by sensory systems (e.g., gustation, chemosensory cells, olfactory epithelium at the gills, chemoreceptors in the antennulae, the olfactory nerve center of the suprapharyngeal ganglion, sensory bristles, and aesthetascs, for example) [50,84,85]. However, some contaminants like metals or pesticides can interfere with the sensorial process and affect the related behavioral response [50,86,87,88,89,90]. This interference can be caused by different mechanisms: direct exposure and damage to exposed olfactory neurons or the disruption in the expression of olfactory system-related genes [85,91]. Thus, the interaction of a contaminant with the sensory system of an organism can affect the behavioral response without a direct relationship with its toxicity. This becomes particularly relevant when the scenario of a mixture of pollution is considered, as the presence of one contaminant can interfere with organism’s response regarding another.

Another factor that makes it difficult to link repellency and toxicity is related to any stimulative or lethargic effects. Some contaminants cause overexcitement in organisms, which indicates toxicity, but that could favor organisms fleeing from contamination. On the other hand, this same contaminant, depending on the concentrations, may induce a lethargic state, which might prevent escape [92]. An interesting pattern was observed in tadpoles exposed to a 2,4-D-based herbicide [66]: the distance-travelled response was not altered, while the speed of response to a stimulus was reduced (both using forced exposure); however, the avoidance in a non-forced system was evident at the lowest concentrations, but less marked at the highest. In another study with tadpoles exposed to sublethal concentrations of copper sulfate and ammonium nitrate, impairments in some behavioral indices (response to stimuli, distance moved and type of movement) were observed, leading to a reduction in the ability to escape [93]. Lethargy has also been observed in tadpoles exposed to copper, where at 200 µg/L the avoidance reached 80% but decreased due to moribundity [63]. Additionally, in tadpoles exposed to mining tailings, there was a tendency for individuals to avoid low concentrations, but not the compartments with highest levels of tailings [68]. In a study with the marine shrimps *L. vannamei* and *P. varians* (Redondo et al. *unpublished data*), it was observed that both are able to avoid toxic copper concentrations when exposed to a gradient; however, whereas *L. vannamei* showed signs of overexcitement when it was in a forced exposure, *P. varians* clearly displayed lethargy.

The best way to verify the relationship between toxicity and repellency is to compare the mortality and repellency data of different chemicals for the same species and then to verify whether the repellency levels of the compounds (from less to more toxic) is related to the toxicity levels. After a bibliographic search, we found little data on toxicity in forced systems and repellency in non-forced multi-compartmented systems that could be compared. However, it was found for: the saltmarsh shrimp *P. varians* (exposed to copper, galaxolide, tonalide, and triclosan), the amphibian *L. catesbeianus* (exposed to abamectin, copper, diuron and 2,4-D), and the freshwater fish *D. rerio* (exposed to Ag-NPs, copper, glyphosate, and pyrimethanil) and *P. reticulata* (exposed to atrazine, bisphenol, copper, and triclosan) (Table 1). Although we tried to consider data published for the same species, in the case of the shrimps, toxicity data for copper and triclosan were taken from other species (see details in Table 1). Before reaching a conclusion on the data, it is important to consider that ecotoxicity results may vary depending on the life stage of the organisms, the culture medium, the environmental conditions during experiments, etc. [94,95]. Therefore, comparisons of the results from different studies should be made with caution.

For the saltmarsh shrimp *P. varians*, copper seems to be the least toxic, but the most repellent contaminant. On the other hand, triclosan follows a pattern of lower lethal toxicity and lower repellency. For the two fragrances, galaxolide seems to be highly repellent and have a low toxicity, whereas tonalide seems to present a potential toxicity very similar to its repellency (Table 1).

In the case of the amphibian *L. catesbeianus*, the pesticide abamectin was the most toxic and the second most repellent contaminant and, following a similar pattern, 2,4-D was the least toxic and least repellent chemical. Diuron deserved special attention because it presented a very high repellency, but low lethal toxicity (Table 1). In spite of this apparent low toxicity of diuron, neurological effects associated with acetylcholinesterase (AChE) activity were observed in the fish *Carassius auratus* exposed at 50 µg/L, but not at 5 µg/L [96] which was the concentration at which the avoidance of tadpoles of *L. catasbeianus* was maximum (around 90%) [67]. Interestingly, the avoidance reduced to 20% at 10 µg/L, which indicates that the increase of diuron concentration caused a reduction in the ability to avoid it [67].

For the fish *D. rerio*, Ag-NPs, copper and glyphosate presented a similar repellency, but in terms of toxicity, this similarity was observed only between copper and glyphosate; Ag-NPs seem to present a lower toxicity. Pyrimethanil seems to be the least toxic and repellent chemical among them (Table 1); in spite of this, the risk cannot be neglected, since sub-lethal effects may be recorded at lower concentrations (38 µg/L) than the AC_50_ [97]. The effects of glyphosate on zebrafish deserve special attention. Although short (96 h) forced exposure to glyphosate can cause behavioral impairments [98], in a 4 h-non-forced exposure approach, avoidance was time-dependent: an attraction was observed during the first two hours, followed by an avoidance in the remaining time (Mena et al., *unpublished data*). This response could be a clear example of time-dependent behavioral hormesis, as the possible overcompensation presented by glyphosate is clearly time-dependent. The importance of time when assessing behavioral changes (initial stimulation followed by a progressive slowdown in movement) after exposure to contaminants has also been pointed out by Ren et al. [48]. An attraction effect has also been observed for female Japanese quails (*Cortunix japonica*) that preferred glyphosate-based herbicide-contaminated food to the control food [26].

Data for *P. reticulata* show that atrazine seems to have a low toxicity, but can be highly repellent, whereas copper seems to be the most toxic, but less repellent; although the AC_50_ values for copper could also be considered very sensitive. Specifically comparing bisphenol A and triclosan, the acute toxicity of both chemicals is very similar; however, bisphenol A is more repellent (Table 1).

The data presented here perhaps represent an oversimplified estimate about the relationship between toxicity (based on mortality) and repellency. Because repellency was based exclusively on studies performed in multi-compartmented exposure systems simulating a contamination gradient, the amount of data is not robust enough to allow for an extensive and more conclusive comparison. However, the data published by other authors and discussed here provide evidence that toxicity cannot be used as a surrogate for repellency. Therefore, we would like to encourage the use of non-forced, multi-compartmented approaches in order to generate a robust database that would help us to understand this relationship between toxicity and repellency better. In addition, the immediate nature of the avoidance makes its interpretation completely different from a forced and extended exposure.

## 5. The Decision of Avoiding or Not: A Cost-Benefits Balance

Ecological systems are very complex and difficult to simulate reliably under any experimental conditions. Many studies have pointed out how the toxicity of a compound can vary depending on the biotic and abiotic changes in the field and under the experimental conditions [107,108,109,110,111]). Any experimental approach in ecotoxicology could be considered environmentally reductionist, but this does not invalidate the importance of the results in terms of understanding the risk of the contamination to the environment. Even the apparently obvious avoidance response triggered by the repellency of contaminants can change if other environmental factors are included. Recent studies in multi-compartmented exposure systems have tested different scenarios by including other elements to the exposure conditions and to verify whether the magnitude of the avoidance response varies and what the level of importance that contamination might have for the habitat selection processes is. The main results found related to other relevant elements in some of these studies are described below. In spite of the factors described below, other factors such as the light should also be studied to understand how the avoidance response might vary during the circadian cycle for diurnal and nocturnal periods.

### 5.1. Population Density

The effects of density on the avoidance response were tested using the freshwater shrimp *A. desmarestii* exposed to a copper gradient [57]. The authors employed three different population densities (3, 5 and 10 shrimps per compartment representing 0.5, 0.8, and 1.7 organisms per mL) in a multi-compartmented system. Avoidance was dependent on the population density, the higher the density, the lower the avoidance. Although shrimps clearly can detect and avoid copper contamination, the stress produced by a high population density (possible intra-species competition) might potentially reduce or even prevent the displacement of organisms to a less disturbed area. The response to toxicants at the population level, when intraspecific competition is present (high population density), differs from the response at the individual level. This was attested by Liess [112], who found that the direct effects of the toxicant were partly compensated by the indirect reduction in intraspecific competitive pressure, which led to a greater availability of food for those who remained.

### 5.2. Competition

The aim of a study performed by Silva et al. [78] with zebrafish (*Danio rerio*) and guppies (*Poecilia reticulata*) was to assess whether the avoidance of both species was affected by the other. In the monospecies experiments, both species avoided the copper gradient in a very similar way: the range of copper concentrations that triggered avoidance to 20, 50, and 80% of the populations overlapped. However, when both species were tested simultaneously (multispecies test), guppies displaced the zebrafish to concentrations that had previously been avoided by the zebrafish. Changes in the avoidance to copper caused by interspecies interactions were also observed in a study with the shrimp *A. desmarestii* and zebrafish [113]. In the presence of fish, the avoidance by shrimps was lower and time-delayed. Both studies evidence that competition among species can change the avoidance pattern in relation to the response in monospecies tests.

### 5.3. Food

The search for food could easily be considered one of the most important drivers that determine the behavior of organisms, especially in conditions where it is not abundant. Based on this statement, an avoidance study was carried out with the fish tilapia (*Oreochromis* sp.) to understand the relationship between the repellency of effluents from a tuna fish processing plant and the availability of food [72]. Firstly, the tilapia fry detected the gradient of contamination and avoided raw and treated effluents. Secondly, organisms were exposed to a gradient of contamination and food simultaneously, so that the more contaminated the area was, the more food was provided. The results indicated that the fish moved intermittently towards the most contaminated areas to feed, in spite of the threat of toxicity.

In another study performed by Islam et al. [79], the effect of food was assessed in three different approaches: avoidance, recolonization and habitat fragmentation. Differently to the method used by Araújo et al. [72], the zebrafish were exposed to a copper gradient, but food was not introduced as a gradient, in the study by Islam and colleagues. In the approaches of avoidance and recolonization, the food was only available in the last and most contaminated zones, whereas in the approach using a chemical fragmentation of habitat, food was only available after the chemical barrier. Those authors found that food did not stimulate the fish to cross the barrier, probably because the trade-off was not perceived.

### 5.4. Predators and Shelters

In a complex environment, the organisms’ decision to avoid or not a contaminated area might be evaluated according to the costs and benefits provided by the different environmental components. In this sense, a study performed with the freshwater shrimp *A. desmarestii* assessed the importance of three elements (i. contamination by copper, ii. presence of shelter that provided protection and iii. kairomones of trout as a predator signal) in the shrimp’s habitat selection process [59]. When the shrimps were exposed to the three elements individually, the contaminated areas and areas with the presence of trout kairomones were avoided, whereas the zones with shelter were preferred. If the organisms were provided with a choice between a clean area with no protection and a contaminated area with protection, they preferred the clean area in spite of the lack of protection. However, when a predator signal was included in the clean area in that scenario, the shrimps moved towards a moderately contaminated area, avoiding the predation risk and the most contaminated zones. This is clear evidence of the disturbance that contamination might cause in the habitat selection process of this species.

The cost–benefit analysis that the organisms “need to carry out” in the presence of several stressors (predation and toxicants) could lead to unexpected results in a kind of compensatory outcome. That could support the hypothesis of “functional compensation” of stressor effects that has been described when an unexpected outcome occurs in a multiple stressor scenario.

### 5.5. Salinity

Salinity is a factor that deserves special attention, not only due to the salinization of coastal freshwater ecosystems (which causes an osmotic unbalance), but also because it is a global and growing threat that might be amplified by climate and anthropic causes [114] and a potential avoidance trigger for many organisms [54]. For instance, fluctuating salinities in estuarine areas can create a very restrictive environment that requires a high osmoregulation capacity [115], which makes salinity a primary environmental factor determinant for the spatial distribution of species [116,117]. In an experiment, Venâncio et al. [54] showed how the cladocera *D. magna*, the ostracod *Heterocypris incongruens*, the amphibian *Xenopus laevis*, and the fish *D. rerio* detected and avoided increasing salt concentrations at much lower levels than those considered lethally dangerous. By combining salinity with contamination (in this case the insecticide diazinon), Mena et al. [61] observed that the ability of the white leg shrimp *L. vannamei* to avoid diazinon was impaired at a salinity of 30, but not at 10 and 20; at a salinity of 30 a higher effect on osmoregulation was also detected. Although salinity is itself a potential avoidance-driving element, in combination with another avoidable element it can have more serious consequences for organisms, either by potentiating avoidance or even preventing it and causing toxicity [54,61].

## 6. Ecological Improvements by Simulating a Chemically Heterogeneous Environment

All the approaches used in ecotoxicology have advantages and limitations regarding the information provided. If identifying ecological succession in a contaminated ecosystem is very important, it is no less important to understand the mode of action of the chemicals (especially the contaminants of emerging concern) and how genetic and physiological mechanisms are triggered in response to contamination [118]. Apart from this, it is widely recognized that ecological approaches are much less frequent than individual or sub-organism approaches. The multi-compartmented exposure approach simulating chemically heterogeneous scenarios does not definitively solve the problem of the lack of ecological relevance of ecotoxicity tests. The aim of the approach presented here is to provide a complementary approach to how the repellency of contaminants can be assessed. Although we know the intrinsic limitations of this approach, some ecological concepts can be integrated into ecotoxicological studies when the spatial chemical heterogeneity is considered, since that the avoidance response of populations might suppose changes in ecological interactions and, therefore, in the ecosystem’s structure and functioning. Some of the improvements provided by the multi-compartmented exposure approach are discussed below.

### 6.1. Spatial Displacement: Extinction at the Local Level

The study of the repellency of the contaminants in a chemically heterogeneous spatial exposure scenario shifts the paradigm of responses and effects. Assuming that organisms could potentially detect contaminants at levels of risk and, therefore, move to more favorable areas, the concept of the stress associated to toxicity at the individual level would not necessarily be applied. When organisms flee an area, although there seems to be no direct effect on the organisms themselves, the loss of abundance of the population that fled could be a major problem at the ecosystem level [30,51] and trigger indirect effects on other species or alterations of ecosystem’s functions [18]. The analysis of the avoidance response goes beyond the repellency of contaminants or even the ability of organisms to detect them, but it brings ecological implications that could lead to a local reduction in biodiversity that, at the same time, could suppose an increase in the species that are highly tolerant to a specific type of contamination, but probably less tolerant for novel stressors [18]. Other effects include restrictions in habitable areas, changes in the species’ interactions (e.g., trophic relationships) in the avoided ecosystems, alterations to migratory patterns, etc. Although in situ observations of the relation between contamination and restrictions in the habitat use are scarce, some studies have evidenced the effects of contamination on the spatial distribution of fish [28,119,120,121].

Finally, when avoidance is associated with a short-term response that also involves the loss of organisms, such as lethality, avoidance data can be used to predict the immediate decline of a population (PID: Population Immediate Decline); a concept developed by Rosa et al. [51] that has been applied in different studies [62,71,73]. The PID calculated from the integration of avoidance (repellency in a non-forced approach) and mortality (toxicity in a forced approach) could help us to understand to what extent the population will decrease better, by considering the proportion of potential avoiders and the proportion of fatalities expected to occur in the non-avoider population. Local extinction rates are affected by spatial heterogeneity and migration rates [122]. The increase in mortality rates, due to toxicity, together with the increase in emigrant rate, would lead to an increased local extinction rate and reduce the probability of local persistence.

### 6.2. Potential to Predict the (Re)Colonization of Environments

Another interesting concept to be employed in this approach is about (re)colonization and restoration of disturbed environments. Generally, ecotoxicity studies are focused on contaminated environments, and so less attention has been given to ecosystem recovery. The concept of colonization in non-forced exposure studies may be used to understand the threshold of the contamination that allows individuals to move from a clean area to an area with acceptable levels of contamination [79,123]. The idea is to identify the levels of contamination that prevent colonization, a chemical threshold from which colonization is less probable or prevented completely. According to the “avoidance-recolonisation hypothesis” [123], the capacity of an ecosystem to receive individuals could be predicted by avoidance tests as follows: if x% of the population avoids a given level of contamination, it is expected that a proportion of 100-x% of the population colonizes an environment with that level of contamination. However, it is unlikely this relation would be so linear as other factors may affect the decision to avoid or not an area ([18,79] see also the discussion in Section 5 of the current review). Furthermore, the repellency and attraction of the chemicals can present a non-linear pattern due to the hormetic effects [38,124]. We encourage the application of the colonization concept as a measure of an ecosystem’s ability to recover from a disturbance, as well as of the organisms’ emigration/immigration patterns. This approach could provide insights about the species that can (or not) potentially colonize an area, which would allow researchers to predict the ecological implications that colonization might represent to the ecosystem. The multi-compartmented approach is an alternative method that may be used to integrate the conceptual model of an affected community based on the dynamics of invader/remainer/escaper [18].

### 6.3. Chemical Fragmentation of Habitat

Habitat fragmentation occurs as result of a discontinuity of the habitat, generally linked to a physical barrier that isolates populations. However, a habitat can be chemically fragmented if the levels of chemicals present in some areas limit the displacement of organisms, even when there is no physical barrier [120,125]. To our knowledge, the application of the concept of chemical fragmentation of habitat using multi-compartmented exposure systems has only taken place in the studies by Araújo et al. [58,74] and Islam et al. [79]. In Araújo et al. [74], the authors showed that the fish *P. reticulata* avoided the herbicide atrazine and that the concentration (105 µg/L), eliciting an avoidance of 80%, produced an isolation of around 50% of the population. A similar study using the fish *D. rerio* and copper showed that a concentration (90 µg/L) that elicited an avoidance to 50% of the population led to the isolation of 41% of that population [79]. This percentage did not vary when food was provided on the clean side, probably because the organisms could not perceive it until after crossing the chemical barrier. The chemical fragmentation of the habitat was also tested with samples of water and sediment from the river Guadalete (Southwest of Spain) [58]. The authors took samples from different parts of the river and simulated the sampled points in a multi-compartmented system. The experimental results evidenced that contamination in both water and sediment might potentially cause a population isolation of the freshwater shrimp *A. desmarestii* that was unable to cross the chemical barrier formed by the most contaminated samples [58]. The possibility of using the concept of chemical barrier in ecotoxicological studies would help to understand another role of contamination that disturbs the ecosystem’s equilibrium and interrupts spatial continuity. The chemical fragmentation of a habitat is a theme that deserves special attention because environmental restrictions can lead to local extinctions, due to the reduction of individuals causing genetic erosion, which may increase the vulnerability of the population [125,126].

In isolated populations, due to habitat restrictions, the risk of extinction is increased and can occur in two main ways (following the extinction vortex model by Gilpin [127]): (i) the allogenic vortex driven by change in the environment (pollution) and (ii) autogenic vortex, driven by population genetics (population isolation leads to a smaller gene pool and the loss of adaptability to environmental change/disturbance).

### 6.4. Habitat Connectivity, Metapopulation, Metacommunity, and Meta-Ecosystem

One of the indirect consequences of contamination is the loss of continuity of habitats that present a patchy distribution in terms of environmental quality. In some circumstances, environmental heterogeneity induces populations to form a spatial arrangement such as metapopulation, moving among habitats with different conditions and, therefore, transferring matter and energy among them (meta-ecosystem) [5,128,129]. Initially, the strategy of avoiding contamination may be successful environmentally due to the absence of stress at the individual level as previously discussed. However, the consequences of this change in the arrangement of the populations could affect the ecosystem where the organisms moved to, since these new individuals might cause some changes in the ecological relationships in that ecosystem [18,130].

The loss of individuals due to avoidance can have important ecological implications on the structure and functioning of ecosystems. The avoidance by the most sensitive species (regarding the ability to detect a contaminant) might lead to indirect effects on other more resistant species (and even on species that cannot flee), due to creating an imbalance in the community, affecting not only the species with which the avoiders have a direct relationship (e.g., predator–prey relationship), but also ecological interactions and even biogeochemical cycles (e.g., energy flow and nutrient cycling) due to the reduction or absence of key species for some ecosystem functions. In this sense, it would be interesting to know how the avoidance of organisms belonging to different trophic levels influences the functioning of the ecosystem, although these ecological questions might require a different experimental approach.

The use of systems such as HeMHAS favors an understanding of the importance that uncontaminated zones might represent as potential areas (refuges) to protect populations against contamination and alleviate individuals from a continuous stress [39]. These authors showed experimentally that in patchy contamination scenarios, the shrimps *A. desmarestii* could present a distribution partially conditioned by copper contamination and dependent on the presence of clean areas in the environment. The complexity of the experimentation systems, such as HeMHAS [52], is crucial to simulate more chemically complex scenarios and understand a little more about the consequences caused by contamination concerning the spatial distribution of organisms and the probability of populations persisting in spite of disturbance [131]. Gilarranz and colleagues showed how a system simulating patches with different levels of disturbance could help to elucidate the effect of the discontinuity of habitats on the maintenance of the populations and how the increase in the disturbance could increase the probability of extinction [131]. Thus, within a connected and heterogeneous landscape, two important questions need to be answered: i. how determinant the differences in the ability to avoid among species of a metacommunity are to the structure the local communities and ii. to what extent the behavioral traits related to avoiding or not contamination could be explained by genetic differences and sensory abilities?

## 7. Final Remarks

As discussed by Ågerstrand et al. [12], the use of ecotoxicological data in regulatory assessments have been based on endpoints such as mortality, growth, reproduction, and development, basically because such responses can lead to population decline. Although this simplifies the application of ecotoxicity tests for a regulatory basis, no other evidence of stress (either to biochemical stress or behavioral alterations) is considered. However, authors and organizations are requesting the inclusion of tests with a higher ecological relevance in the risk assessment of chemical substances [132,133,134]. From a conceptual point of view, these endpoints (mortality, growth, reproduction, and development) are directly related to the toxicity of chemicals, following the cascade of effects [40,41] that are triggered when organisms are exposed to contaminants continuously (cascade of effects related to toxicity). Considering that some organisms cannot avoid contamination (cascade of effects related to repellency), either because of their inability to move or because the spatial scale of contamination is spread beyond the area to which they could move, the focus on toxicity is appropriate and ecologically relevant. However, for mobile organisms and in a heterogeneous contamination scenario, the potential repellency of the chemicals should also be considered. The application of this repellency as an endpoint for regulatory application could be easily justified since the evasion of organisms might lead to a population decline at the local level [18,30]. As discussed in the current review, this displacement might cause disturbances at the structural and functional levels, not only in the ecosystems avoided, but also in the alternative chosen one. This approach extends the concept of stress to a level beyond the individual response [1,18], integrating the susceptibility of organisms (that can require adaptation or lead to a loss of the most sensitive species) and environmental vulnerability (see Figure 1 and discussion in [6,7,12,18]). In addition, the repellency response is expected to be immediate (normally after not more than a 12 h exposure) [70], which could help reduce misunderstandings related to a time-delayed effect due to a continuous and extended exposure [70].

Although the theoretical basis that could justify the use of behavioral responses has been recognized [21,135,136], the practical application of this approach is criticized mainly for the lack of standard protocols that could help minimize the errors associated to observation [12]. However, the implementation of automatic systems has contributed to increasing the validity of traditional behavioral responses [12,137]. For tests with aquatic organisms in non-forced multi-compartmented systems some attempts have been made to standardize the procedures, namely with the publication of a standard operating procedure for linear systems [123] and the development of the HeMHAS [52], but much more effort is still required.

The final reason why behavioral ecotoxicology is not employed for a regulatory basis can be sustained by the lack of results using behavioral endpoints. In the case of avoidance measured in a multi-compartmented exposure system, this lack is even greater. Therefore, with this review we have intended not only to demonstrate that repellency can trigger an ecologically relevant response such as spatial avoidance, but also to encourage studies using the non-forced multi-compartmented approach to improve our understanding of the spatial distribution of organisms driven by contamination. The aim of the approach presented here is to integrate Stress Ecology and Landscape Ecology, considering contaminants as one more element of ecosystems, from a more ecological perspective (habitat selection processes and the potential interactions with biotic and abiotic factors to “take the decision” of staying or avoiding a habitat), and broadening the spatial scale (landscape) of the observation, considering not only the contaminated and avoided ecosystem, but also the surrounding areas receiving the avoiders (environmental heterogeneity). Finally, such as indicated in Figure 1, we strongly support the integration of toxicity (when the effects are based on the sensitivity of organisms) and repellency (when the organisms change the habitat selected according to the levels of contamination) to achieve a conceptually broader environmental assessment.

## Figures and Tables

**Table 1:** Comparison of the toxic and repellent potential of different contaminants for four species based on data of toxicity (LC_50_: lethal concentration to 50% of the population; in µg/L) and repellency (AC_50_: concentration eliciting avoidance in 50% of the population; in µg/L).

Species	Contaminant	Toxicity (LC_50_)	Avoidance (AC_50_)	References for Toxicity/Avoidance
*Palaemon varians* (saltmarsh shrimp) ^a^	Copper	660	10.4	[25,99]
	Galaxolide	401	14.1	[62]
	Tonalide	88.1	30.8	[62]
	Triclosan	154	42	[100,101]
*Lithobates catesbeianus* (amphibian) ^b^	Abamectin	138	36	[65]
	Copper	372	101	[63]
	Diuron	31,000	±0.5 ^c^	[67]
	2,4-D	574,000	242 ^d^	[66]
*Danio rerio* (freshwater fish)	Ag-NPs	2900	9.08	[102], Sendra et al. (*unpublished data*)
	Copper	880	16.7	[78,103]
	Glyphosate	620	12.2	[104], Mena et al. (*unpublished data*)
	Pyrimethanil	2850	1100	[71,97]
*Poecilia reticulata* (freshwater fish)	Atrazine	4300	0.065	[74,105]
	Bisphenol A	1660	0.154	[77]
	Copper	348	15.9	[78,106]
	Triclosan	1650	8.04	[73]

^a^: Toxicity data of copper and triclosan were based on the post larvae of *Penaeus monodon* [99] and larvae of *Palaemonetes pugio* [100], respectively. ^b^: Gosner stage 25. ^c^: the AC_50_ value was not provided, but the authors reported an avoidance of around 50% at 0.5 µg/L. ^d^: the AC_50_ value was not provided, but the authors reported an avoidance of around 50% at 242 µg/L.
